# Supplementary material for: Versatile Cell and Animal Models for Advanced Investigation of Lead Poisoning
Source: Biosensors (Basel). 2021 Oct 4;11(10):371. doi: 10.3390/bios11100371 (PMC8533970; doi:10.3390/bios11100371)
Supplement: Supplementary file 1 [file biosensors-11-00371-s001.zip › biosensors-1370929-sup/Supp_info_Manuscript_BIOSENSORS.pdf]

biosensors

MDPI

Article

Versatile Cell and Animal Models for Advanced Investigation of Lead Poisoning

De-Ming Yang [1,2,*] and Yu-Fen Chang [3]

1 Microscopy Service Laboratory, Basic Research Division, Department of Medical Research, Taipei Veterans General Hospital, Taipei, Taiwan; dmyang@vghtpe.gov.tw
2 Institute of Biophotonics, School of Biomedical Science and Engineering National Yang Ming Chiao Tung University, Taipei, Taiwan; yang.deming2021@nycu.edu.tw
3 LumiSTAR Biotechnology, Inc., Taipei City 115, Taiwan; yu-fen.chang@lumistar.com.tw
* Correspondence: dmyang@vghtpe.gov.tw

Additional Figures.

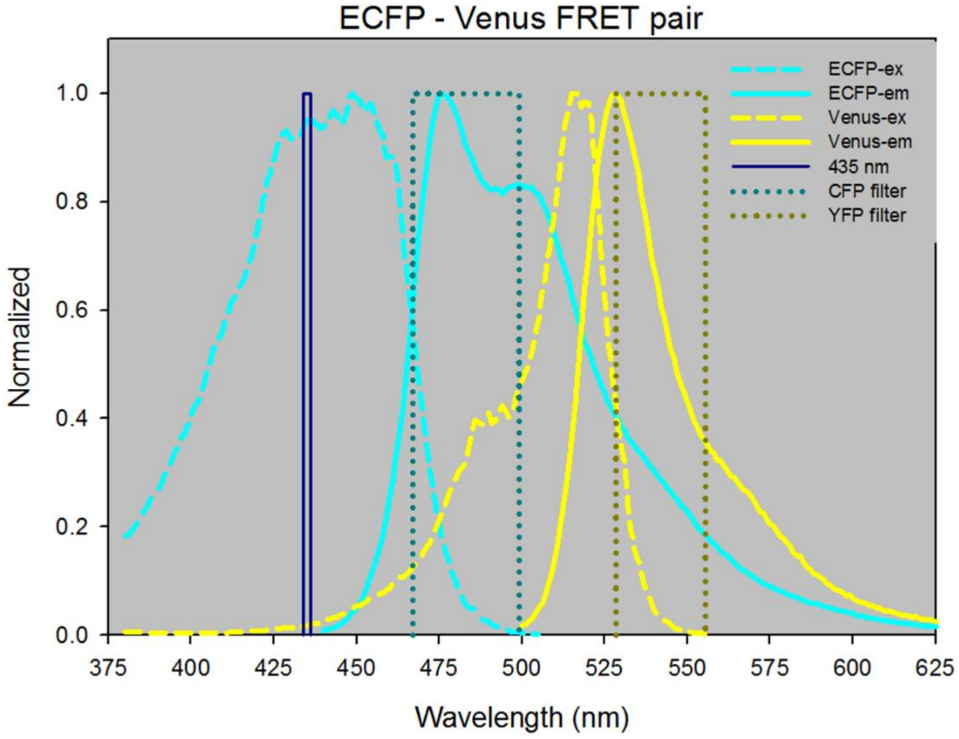

Figure S1. Spectral analysis of the FRET pairs (ECFP and EYFP/Venus) for the considerations of related filter setup. The spectral relationship between donor (ECFP in light blue) excitation (dash line)/emission (solid line) with acceptor (EYFP/Veuns in yellow) excitation (dash line)/ emission (solid line) under the emission and excitation spectrum scan of these FPs. The dark blue rectangle space indicates the excitation laser source for ECFP (435 nm). The dashed rectangle spaces indicate the suggested emission ranges of ECFP (in dark cyan) and those of EYFP/Venus (in dark yellow) to prevent signal cross-over.

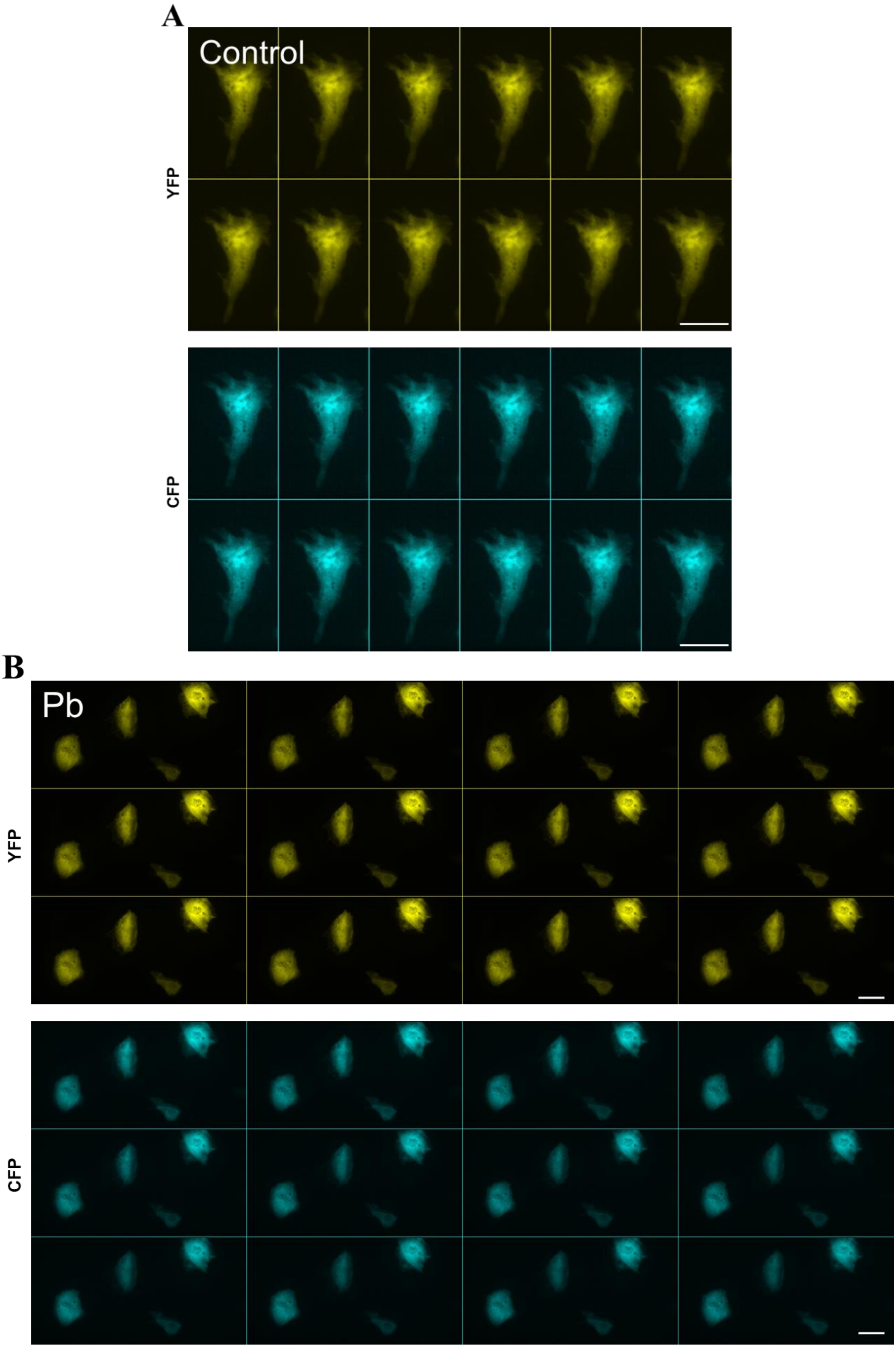

Figure S2. Original data for the assessment of the physiological activity and entry of Pb on iPSC-derived cardiomyocytes (Figure 2). Representative sections of control (Control; A) or in Pb buffer (Pb; B) in YFP and CFP channels are shown. The scale bars are 40 μm.

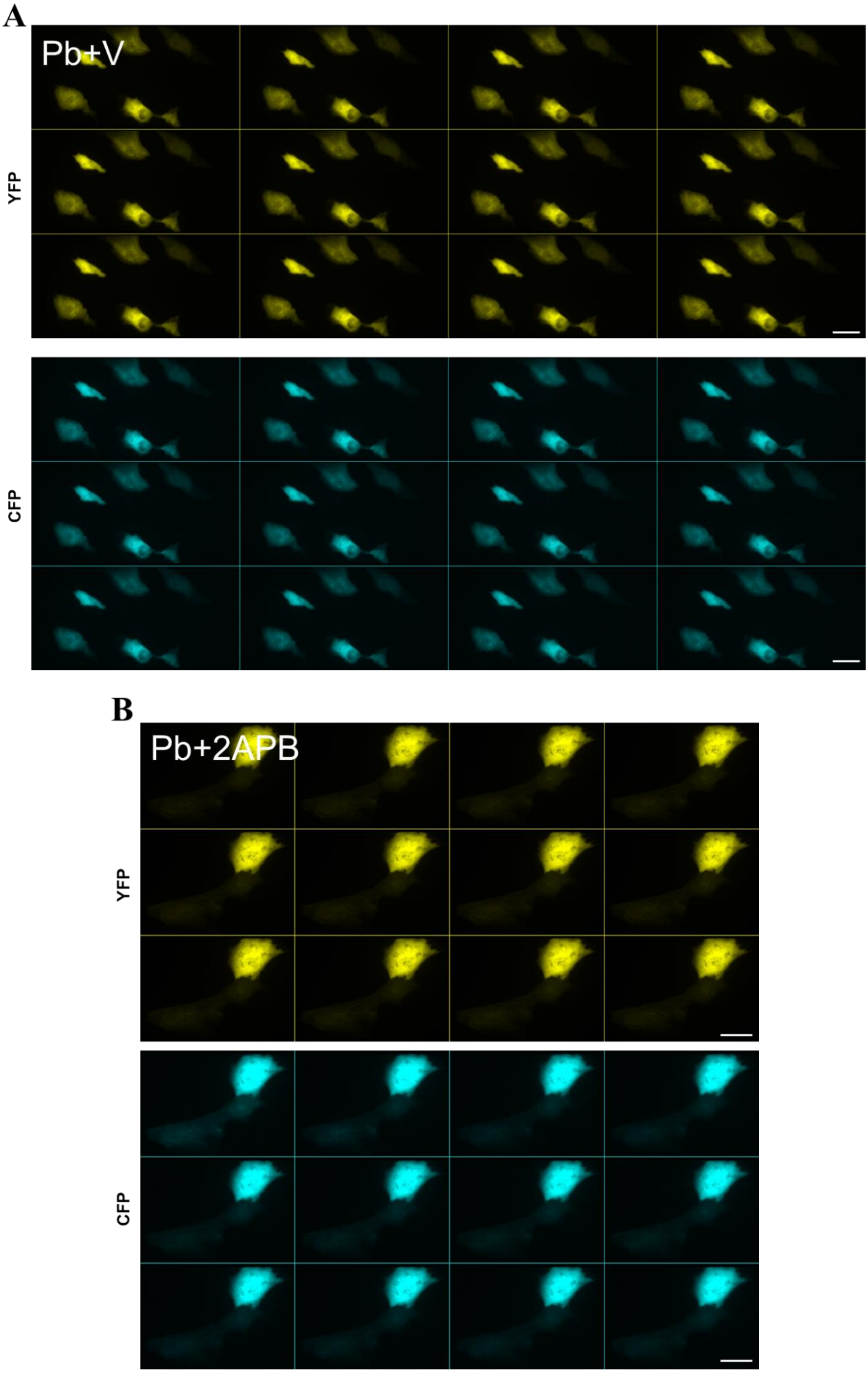

Figure S3. Original data for the assessment of the differential effects of Ca^{2+} blockers on iPSC-derived cardiomyocytes (Figure 3). Representative sections of Pb buffer with verapamil (Pb+V; A) or in Pb buffer with 2-aminoethoxydiphenyl borate (2-APB) (Pb+2APB; B) in YFP and CFP channels are shown. The scale bars are 40 μm.

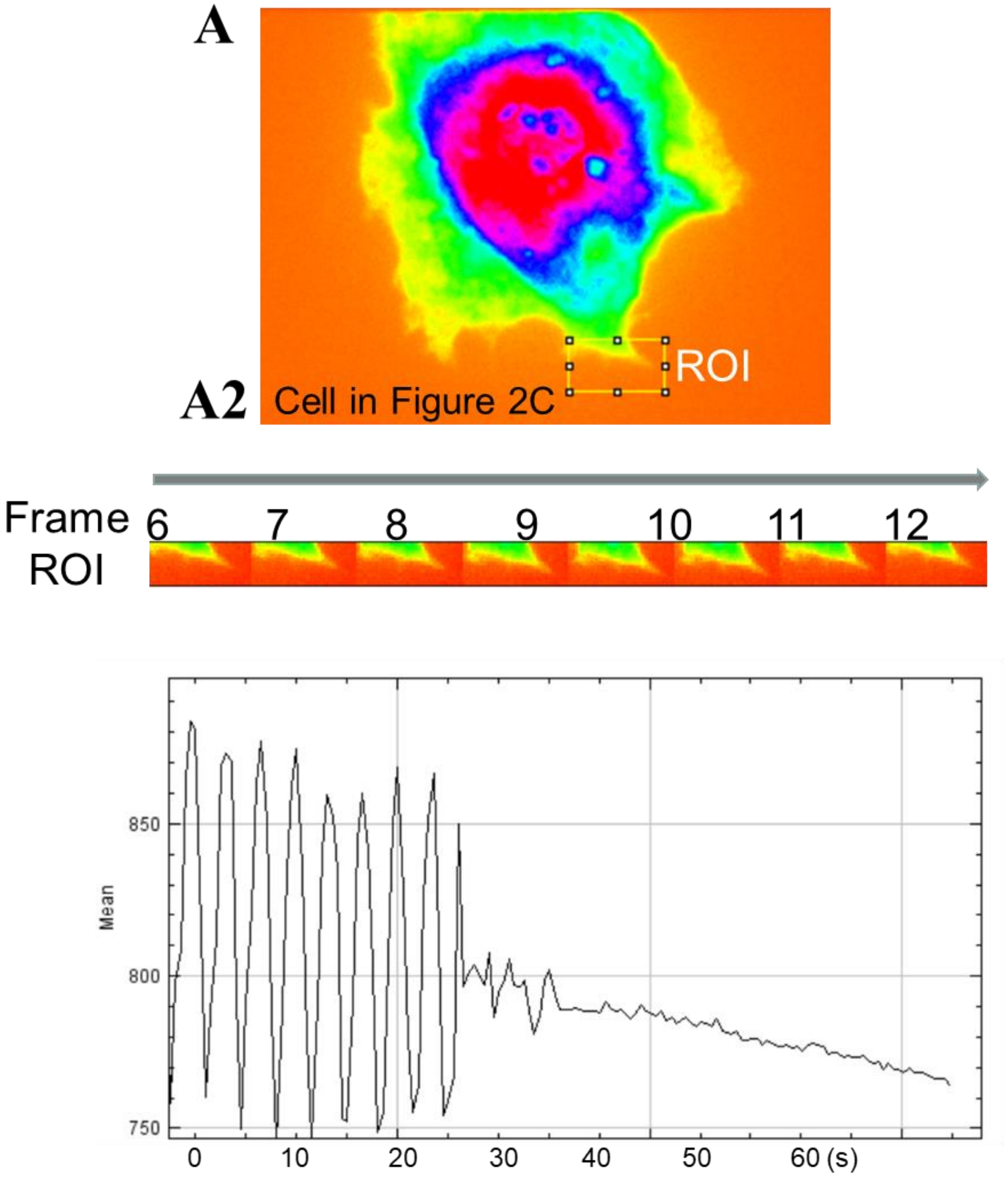

Figure S4. The analysis of beating frequency from the fluorescent recording of iPSC-derived cardiomyocytes. Generally, we put the ROI close to the edge of the cells as shown in A. Contraction of the cells causing the repetitive movement of object shown in ROI (A2) would change the total intensity of ROI as plotted in B.

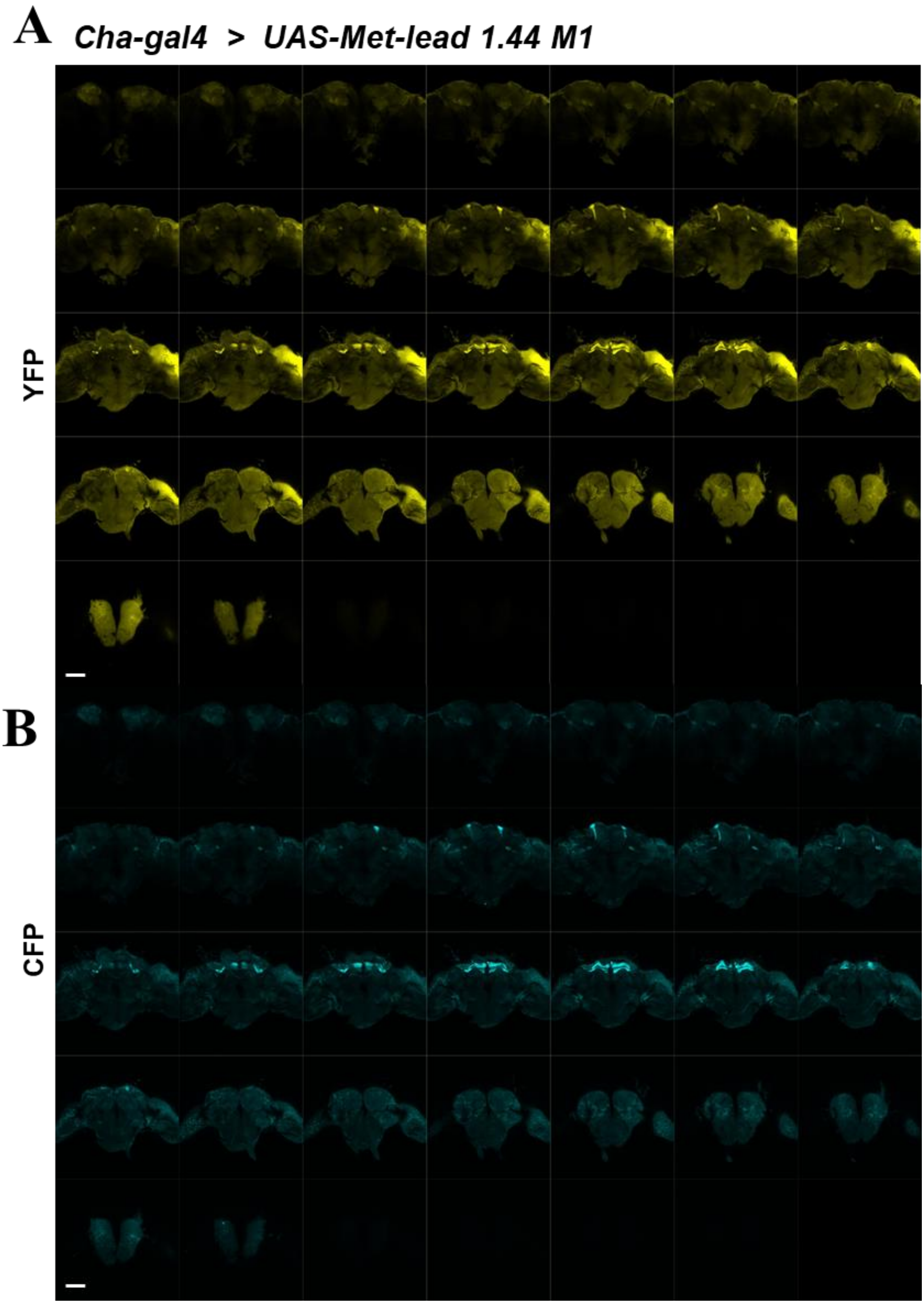
A
Cha-gal4 > UAS-Met-lead 1.44 M1
YFP
B
CFP

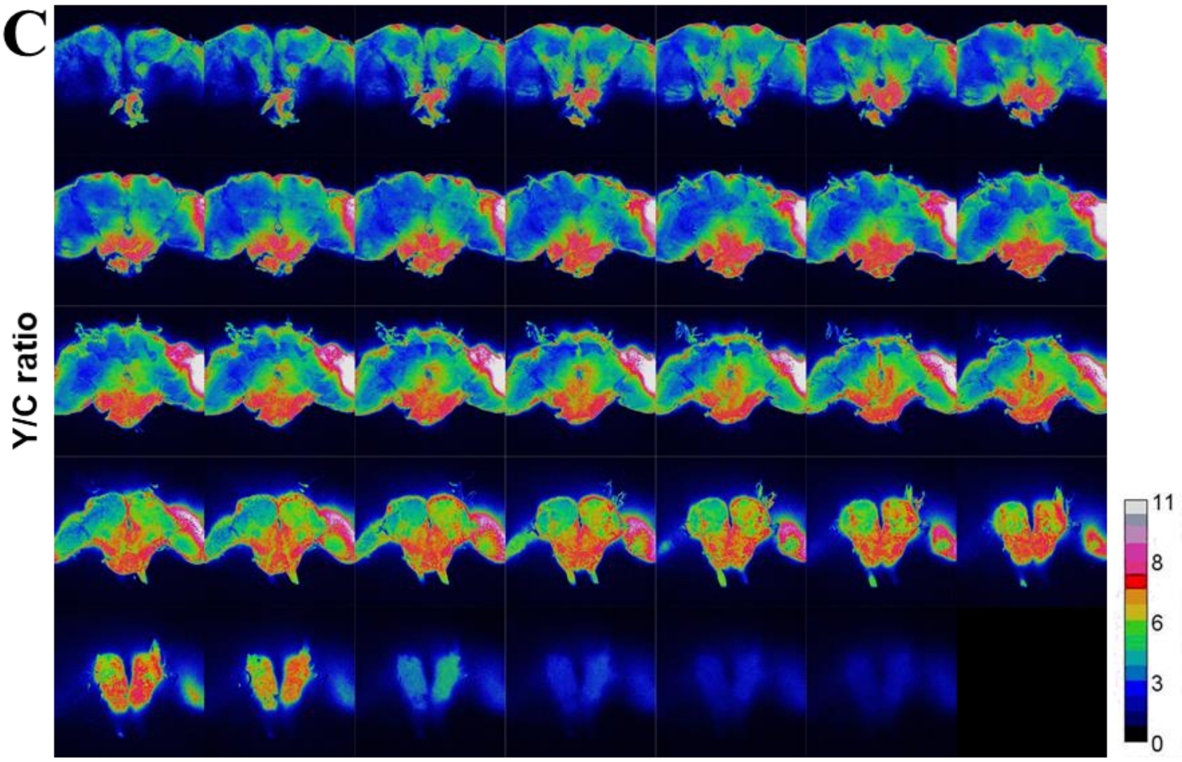

Figure S5. *Ex vivo* Pb biosensing in the adult brain of *Drosophila* (strain *Cha-gal4* > *UAS-Met-lead 1.44 M1*) using high-resolution two-photon FRET ratio imaging. Thirty-four optical sections of brain images in YFP, CFP and Ratio channel are shown in A, B and C respectively. The color bar in C represents ratio values from 0 to 11. The scale bars are 100 μm.

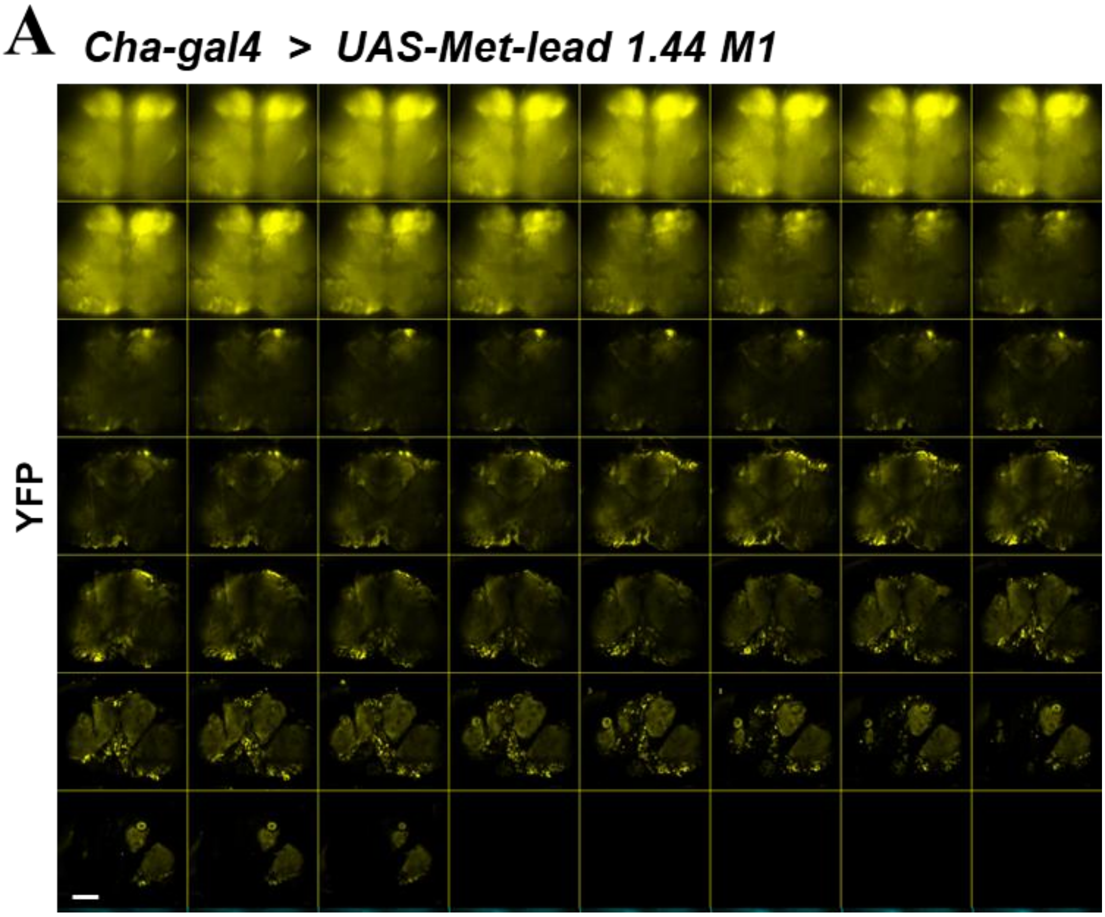

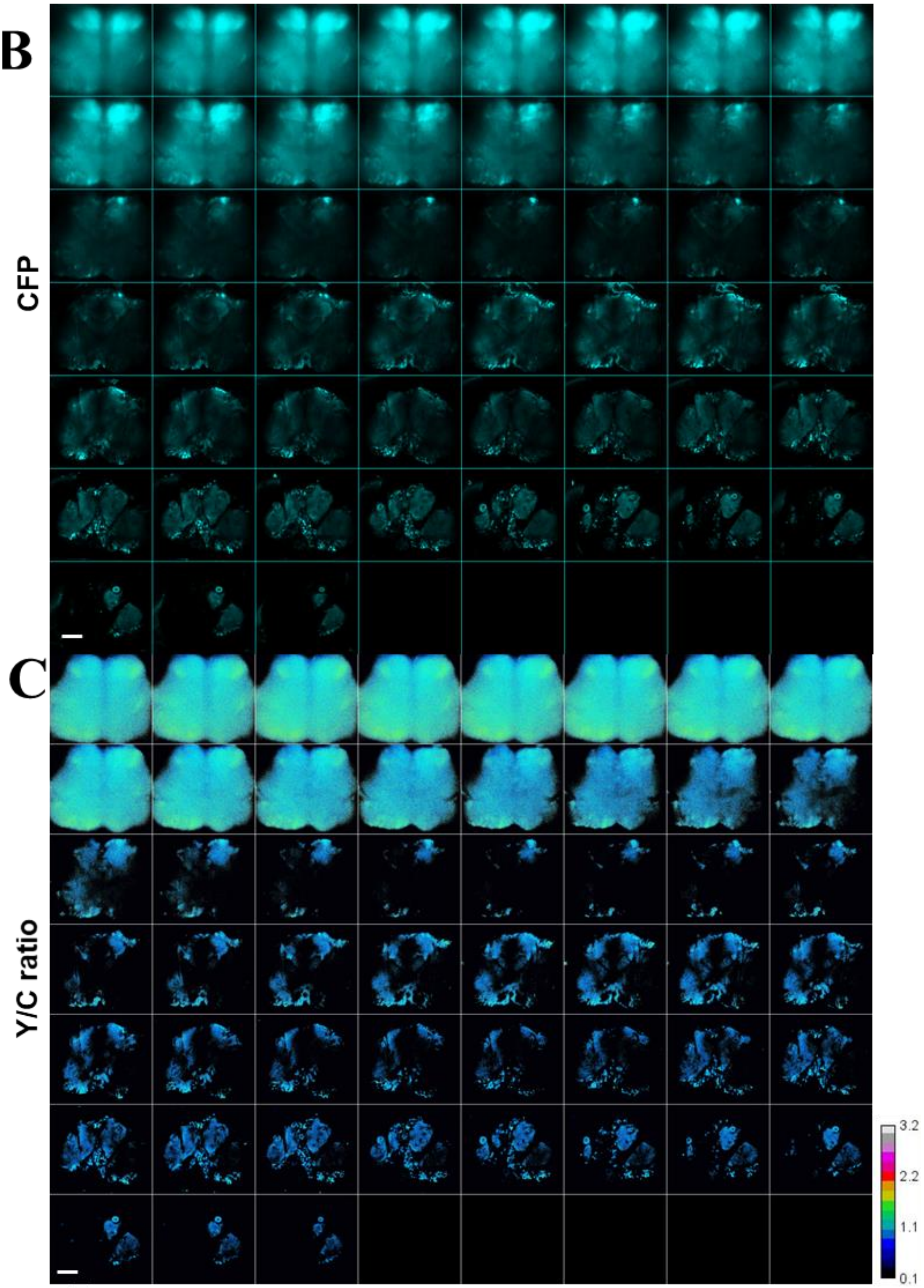

Figure S6. *In vivo* Pb biosensing in the adult brain of *Drosophila* (strain *Cha-gal4 > UAS-Met-lead 1.44 M1*) using high-resolution two-photon FRET ratio imaging. Fifty-one optical sections of brain images in YFP, CFP and Ratio channel are shown in A, B and C respectively. The color bar in C represents ratio values from 0.1 to 3.2. The scale bars are 50 μm.

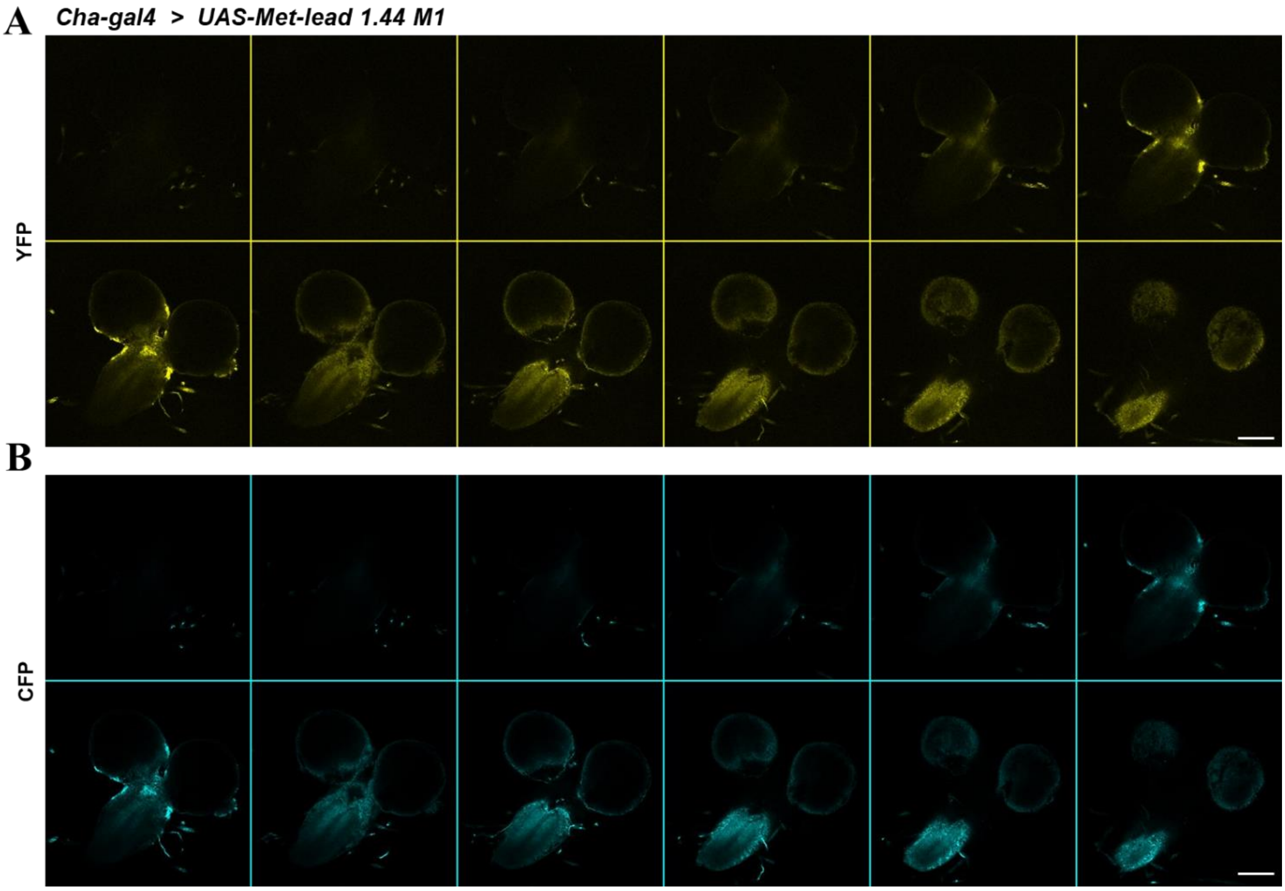

Figure S7. Original data of *in vivo* Pb biosensing in the larval CNS of *Drosophila* for Figure 5. Representative sections of larval CNS in YFP and CFP channels are shown in A and B respectively. The scale bars are 100 μm.
