# Supplementary figures and images for: Versatile Cell and Animal Models for Advanced Investigation of Lead Poisoning
Source: Biosensors (Basel). 2021 Oct 4;11(10):371. doi: 10.3390/bios11100371 (PMC8533970; doi:10.3390/bios11100371)

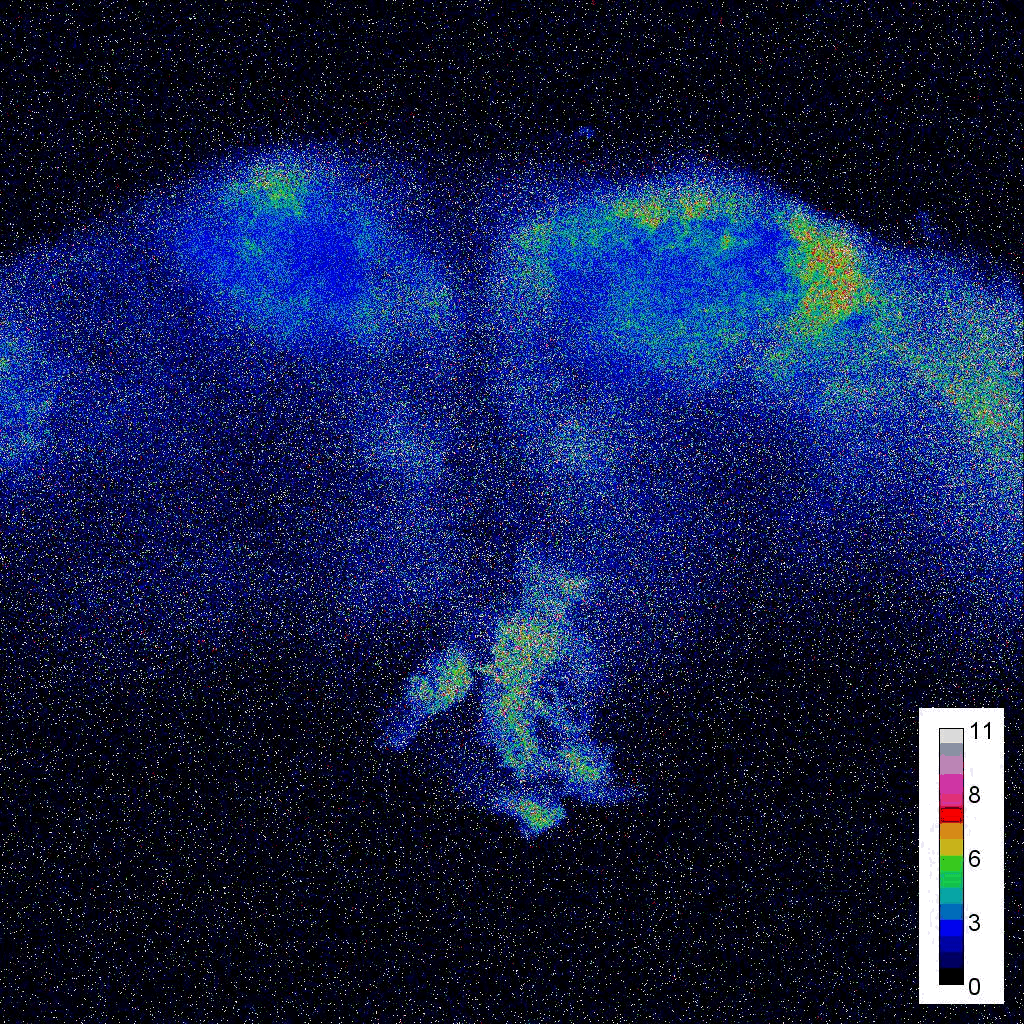

Supplement: Supplementary file 1 [file biosensors-11-00371-s001.zip › biosensors-1370929-sup/Video S2_Figure_S5_RATIO.gif]

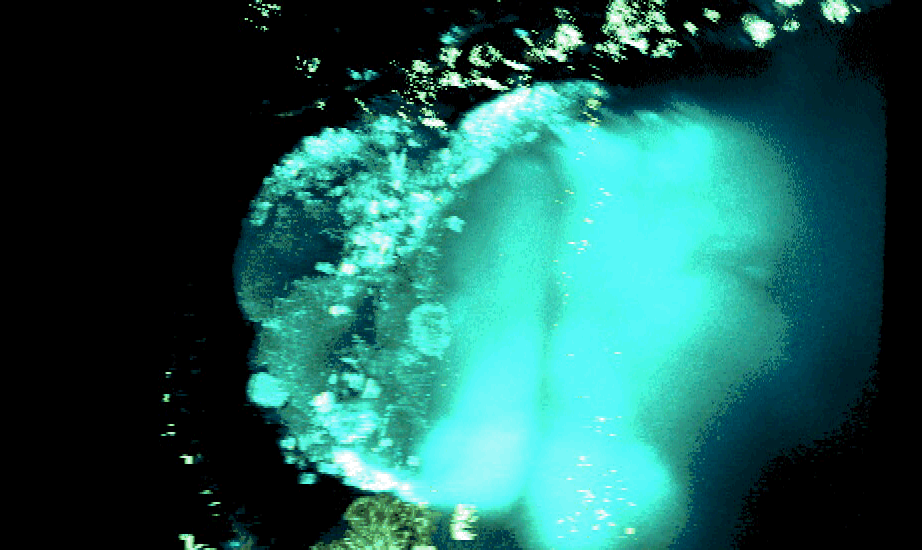

Supplement: Supplementary file 1 [file biosensors-11-00371-s001.zip › biosensors-1370929-sup/Video S3_Figure_S6_3D.gif]

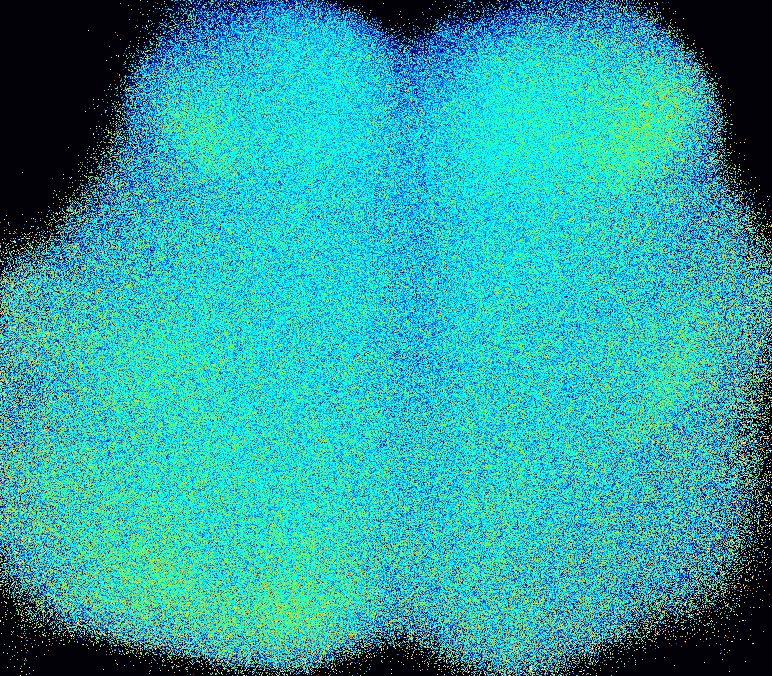

Supplement: Supplementary file 1 [file biosensors-11-00371-s001.zip › biosensors-1370929-sup/Video S4_Figure_S6_RATIO.gif]

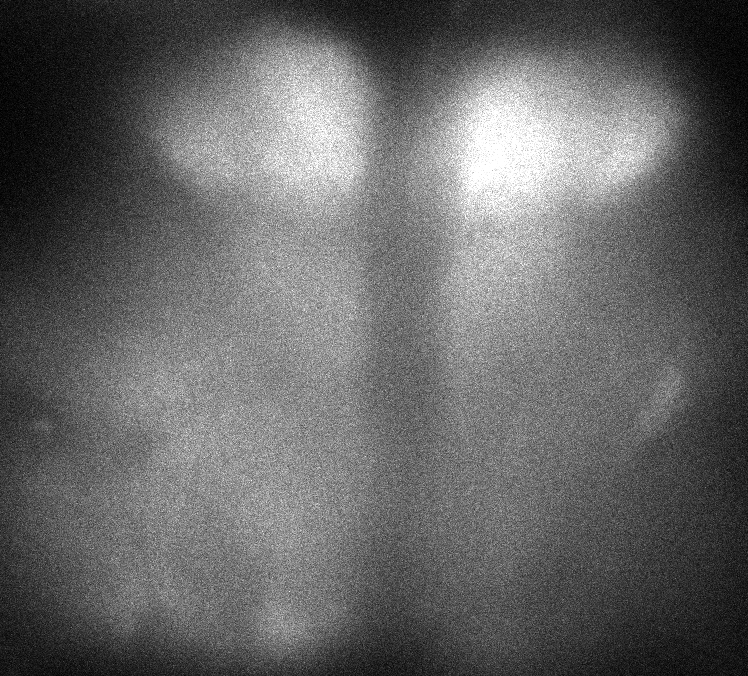

Supplement: Supplementary file 1 [file biosensors-11-00371-s001.zip › biosensors-1370929-sup/Video S5_Figure_S6_YFP.gif]
